# Supplementary material for: Dynamic control of DNA condensation
Source: Nat Commun. 2024 Mar 1;15:1915. doi: 10.1038/s41467-024-46266-z (PMC10907372; doi:10.1038/s41467-024-46266-z)
Supplement: Supplementary file 1 — Supplementary Information [file 41467_2024_46266_MOESM1_ESM.pdf]

Supplementary Information File
Dynamic control of DNA condensation

Siddharth Agarwal[1,2], Dino Osmanovic[1], Mahdi Dizani[1], Melissa A. Klocke[1] and Elisa Franco[1,2,*]
[1] Department of Mechanical and Aerospace Engineering, University of California at Los Angeles
[2] Bioengineering, University of California at Los Angeles

* Corresponding author: efranco@seas.ucla.edu

1. Supplementary Tables 2
   DNA nanostar variants 2
   Modified nanostars with invader and anti-invader variants 4
   Orthogonal nanostars (Fig. 6) 7
2. Supplementary Figures 8
   2.1 Addition of a non-complementary invader sequence 8
   2.2 Nanostars with different arm length 9
   2.3 Six arm nanostars with adjacent and staggered invasion points after 24 hours 10
   2.4 Orthogonal DNA condensates 11
   2.5 Invasion and anti-invasion of orthogonal 6-nt NS at RT 11
   2.6 Excess of anti-invader limits droplet regrowth due to weak interactions with the nanostar sticky-ends. 12
   2.7 Condensate regrowth in 4 arm NS with addition of more than one Anti-invader 13
   2.8 Annealing nanostars in the presence of invader 14
   2.9 Annealing nanostars at different concentrations (no invader) 17
3. Supplementary Notes 20
   Supplementary Note 1: Mean Field Theory Model 20
   Supplementary Note 2: Impact of Reverse Reactions 23
   Supplementary Note 3: Other features 24
4. Supplementary References 26

1. Supplementary Tables

The sequences of the oligonucleotide strands were designed using NUPACK. We include two spacer bases (TT) at the center of the junction of each nanostar. All sequences in the tables below are listed 5' to 3'.

DNA nanostar variants

Supplementary Table 1: 3-arm – 16 bp arm	
Y1_4_16	GCGCCAGTGAGGACGGAAGTTTGTCGTAGCATCGCACC
Y2_4_16	GCGCCAACCACGCCTGTCCATTACTTCCGTCCTCACTG
Y3_4_16	GCGCGGTGCGATGCTACGACTTTGGACAGGCGTGGTTG
Y1_0_16_cy3	cy3-CAGTGAGGACGGAAGTTTGTCGTAGCATCGCACC

Supplementary Table 2: 3-arm – 16 bp arm - TATA sticky end	
Y1_4_16_TATA	TATACAGTGAGGACGGAAGTTTGTCGTAGCATCGCACC
Y2_4_16_TATA	TATACAACCACGCCTGTCCATTACTTCCGTCCTCACTG
Y3_4_16_TATA	TATAGGTGCGATGCTACGACTTTGGACAGGCGTGGTTG
Y1_0_16_cy3	cy3-CAGTGAGGACGGAAGTTTGTCGTAGCATCGCACC

Supplementary Table 3: 3-arm – 8 bp arm	
Y1_4_8	GCGCCAGTGAGGTTGTCGTAGC
Y2_4_8	GCGCCCTGTCCATTCCTCACTG
Y3_4_8	GCGCGCTACGACTTTGGACAGG
Y1_0_8_cy3	cy3-CAGTGAGGTTGTCGTAGC

Supplementary Table 4: 3-arm – 24 bp arm	
Y1_4_24	GCGCCAGTGAGGACGGAAGTGAAGGAACTTGTCGTAGCATCGCACCGACAAAGC
Y2_4_24	GCGCGTCGCATCCAACCACGCCTGTCCATTGTTCCTTCACTTCCGTCCTCACTG
Y3_4_24	GCGCGCTTTGTCGGTGCGATGCTACGACTTTGGACAGGCGTGGTTGGATGCGAC
Y1_0_24_cy3	cy3-CAGTGAGGACGGAAGTGAAGGAACTTGTCGTAGCATCGCACCGACAAAGC

Supplementary Table 5: 3-arm – 32 bp arm	
Y1_4_32	GCGCCAGTGAGGACGGAAGTGAAGGAACTCTCCGCGTTGTCGTAGCATCGCACCGACAAAGCGAACACGT
Y2_4_32	GCGCGCCTCTGTGTCGCATCCAACCACGCCTGTCCATTCGCGGAGAGTTCCTTC ACTTCCGTCCTCACTG
Y3_4_32	GCGCACGTGTTCGCTTTGTCGGTGCGATGCTACGACTTTGGACAGGCGTGGTTGGATGCGACACAGAGGC
Y1_0_32_cy3	cy3-CAGTGAGGACGGAAGTGAAGGAACTCTCCGCGTTGTCGTAGCATCGCACCGACAAAGCGAACACGT

Supplementary Table 6: 4-arm – 16 bp arm	
Y1_4_16	GCGCCAGTGAGGACGGAAGTTTGTCGTAGCATCGCACC
Y2_4_16	GCGCCAACCACGCCTGTCCATTACTTCCGTCCTCACTG
F3_4_16	GCGCCCATGGTCCCAAGTGATTTGGACAGGCGTGGTTG
F4_4_16	GCGCGGTGCGATGCTACGACTTTCACTTGGGACCATGG

Supplementary Table 7: 6-arm – 16 bp arm	
Y1_4_16	GCGCCAGTGAGGACGGAAGTTTGTCGTAGCATCGCACC
Y2_4_16	GCGCCAACCACGCCTGTCCATTACTTCCGTCCTCACTG
F3_4_16	GCGCCCATGGTCCCAAGTGATTTGGACAGGCGTGGTTG
P4_4_16	GCGCCTCAGAGAGGTGACAGTTTCACTTGGGACCATGG
S5_4_16	GCGCGCTGGACTAACGGAACTTCTGTCACCTCTCTGAG
S6_4_16	GCGCGGTGCGATGCTACGACTTGTTCCGTTAGTCCAGC

Modified nanostars with invader and anti-invader variants

Sticky ends are underlined on strands assembling into nanostars.
Toeholds for invasion are indicated in **bold**.

Supplementary Table 8: Invaders and Anti-invaders for 3-arm – 16 bp arm	
I_Y2_16_0toe	TGGTTGGCGC
I_Y2_16_3toe	TGGTTGGCGC**TGA**
I_Y2_16_5toe	TGGTTGGCGC**TGATA**
Y2_4_16_I0	GCGCTGATAGGCAATGC
Y2_4_16_I3	TTGGCGC**TGATAGG**CAATGC
Y2_4_16_I6	TGGTTGGCGC**TGATAGG**CAATGC
Y2_4_16_I9	GCGTGGTTGGCGC**TGATAGG**CAATGC
Y2_4_16_AI0	GCATTC**CCTATCA**GCGC
Y2_4_16_AI3	GCATTC**CCTATCA**GCGCCAA
Y2_4_16_AI6	GCATTC**CCTATCA**GCGCCAACCA
Y2_4_16_AI9	GCATTC**CCTATCA**GCGCCAACCACGC
Y2_4_16_I_AI	**CCTATCA**GCGCCAACCACGCCTGTCCATTACTTCCGTCCTCACTG

Supplementary Table 9: Invaders and Anti-invaders for 4-arm – 16 bp arm	
Y2_4_16_I_AI	**CCTATCA**GCGCCAACCACGCCTGTCCATTACTTCCGTCCTCACTG
Y2_4_16_I6	TGGTTGGCGC**TGATAGG**CAATGC
Y2_4_16_AI6	GCATTC**CCTATCA**GCGCCAACCA
F3_4_I_AI	**GATGTCG**GCGCCCATGGTCCCAAGTGATTTGGACAGGCGTGGTTG
I6_F3_4	CCATGGGCGC**CGACATC**TAAACG
AI6_F3_4	CGTTTA**GATGTCG**GCGCCCATGG

Supplementary Table 10: Invaders and Anti-invaders for 6-arm – 16 bp arm	
Y2_4_16_I_AI_7toe	**CCTATCA**GCGCCAACCACGCCTGTCCATTACTTCCGTCCTCACTG
Y2_4_16_I_AI_5toe	**TATCA**GCGCCAACCACGCCTGTCCATTACTTCCGTCCTCACTG
Y2_4_16_I_AI_3toe	**TCA**GCGCCAACCACGCCTGTCCATTACTTCCGTCCTCACTG
Y2_4_16_I6	TGGTTGGCGC**TGATAGG**CAATGC
Y2_4_16_AI6	GCATTC**CCTATCA**GCGCCAACCA
F3_4_I_AI	**GATGTCG**GCGCCCATGGTCCCAAGTGATTTGGACAGGCGTGGTTG
I6_F3_4	CCATGGGCGC**CGACATC**TAAACG
AI6_F3_4	CGTTTA**GATGTCG**GCGCCCATGG
P4_4_I_AI	**TCAGTCC**GCGCCTCAGAGAGGTGACAGTTTCACTTGGGACCATGG
I6_P4_4	TCTGAGGCGC**GGACTGA**CACGAC
AI6_P4_4	GTCGTG**TCAGTCC**GCGCCTCAGA

Supplementary Table 11: Invaders and Anti-invaders for 3-arm – 8 bp arm	
Y2_8_I_AI_7toe	**CCTATCA**GCGCCCTGTCCATTCCTCACTG
Y2_8_I_AI_5toe	**TATCA**GCGCCCTGTCCATTCCTCACTG
Y2_8_I_AI_3toe	**TCA**GCGCCCTGTCCATTCCTCACTG
I_Y2_8_3toe	GACAGGGCGC**TGA**

Supplementary Table 12: Invaders and Anti-invaders for 3-arm – 24 bp arm	
Y2_24_I_AI_7toe	**CCTATCA**GCGCGTCGCATCCAACCACGCCTGTCCATTGTTCCTTCACTTCCGTCCTCACTG
Y2_24_I_AI_5toe	**TATCA**GCGCGTCGCATCCAACCACGCCTGTCCATTGTTCCTTCACTTCCGTCCTCACTG
Y2_24_I_AI_3toe	**TCA**GCGCGTCGCATCCAACCACGCCTGTCCATTGTTCCTTCACTTCCGTCCTCACTG
I_Y2_24_3toe	TGCGACGCGC**TGA**

Supplementary Table 13: Invaders and Anti-invaders for 3-arm – 32 bp arm	
Y2_32_I_AI_7toe	**CCTATCA**GCGCGCCTCTGTGTCGCATCCAACCACGCCTGTCCATTCGCGGAGAGTTCCTTCACTTCCGTCCTCACTG
Y2_32_I_AI_5toe	**TATCA**GCGCGCCTCTGTGTCGCATCCAACCACGCCTGTCCATTCGCGGAGAGTTCCTTCACTTCCGTCCTCACTG
Y2_32_I_AI_3toe	**TCA**GCGCGCCTCTGTGTCGCATCCAACCACGCCTGTCCATTCGCGGAGAGTTCCTTCACTTCCGTCCTCACTG
I_Y2_32_3toe	AGAGGCGCGC**TGA**

Orthogonal nanostars (Fig. 6)

Supplementary Table 14: Nanostar 1	
Orange_6bSE_Y1	GGATCCCAGTGAGGACGGAAGTTTGTCGTAGCATCGCACC
Orange_6bSE_Y2_I_AI	**CCTATCA**GGATCCCAACCACGCCTGTCCATTACTTCCGTCCTCACTG
Orange_6bSE_Y3	GGATCCGGTGCGATGCTACGACTTTGGACAGGCGTGGTTG
Orange_6bSE_Y2_I6	TGGTTGGGATCC**TGATAGG**CAATGC
Orange_6bSE_Y2_AI6	GCATTC**CCTATCA**GGATCCCAACCA
cy3_Orange_6bSE_Y1	cy3-CAGTGAGGACGGAAGTTTGTCGTAGCATCGCACC

Supplementary Table 15: Nanostar 2	
Green_6bSE_Y1	TGCGCAGAAGGAACTCTCCGCGTTGACAAAGCGAACACGT
Green_6bSE_Y2_I_AI	**AATCGGA**TGCGCAGCCTCTGTGTCGCATCTTCGCGGAGAGTTCCTTC
Green_6bSE_Y3	TGCGCAACGTGTTCGCTTTGTCTTGATGCGACACAGAGGC
Green_6bSE_Y2_I6	AGAGGCTGCGCA**TCCGATT**AGATTC
Green_6bSE_Y2_AI6	GAATCT**AATCGGA**TGCGCAGCCTCT
fam_Green_6bSE_Y1	6fam-GAAGGAACTCTCCGCGTTGACAAAGCGAACACGT

2. Supplementary Figures

Throughout this section, the word nanostars is abbreviated as “NS”.

2.1 Addition of a non-complementary invader sequence

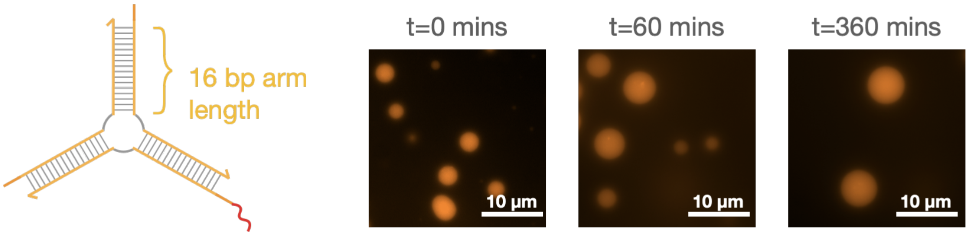

Supplementary Figure 1: Addition of a non-complementary invader sequence has no effect on the condensates. Toeholded 3 arm NS (16 bp arms, 7nt toehold) continue to condense into droplets if a non complementary invader is added. Time t=0 is 30 min after annealing (27° C); [NS] = 5 μM, scramble sequence (Green_6bSE_Y2_AI6) concentration 5 μM (1X NS).

2.2 Nanostars with different arm length

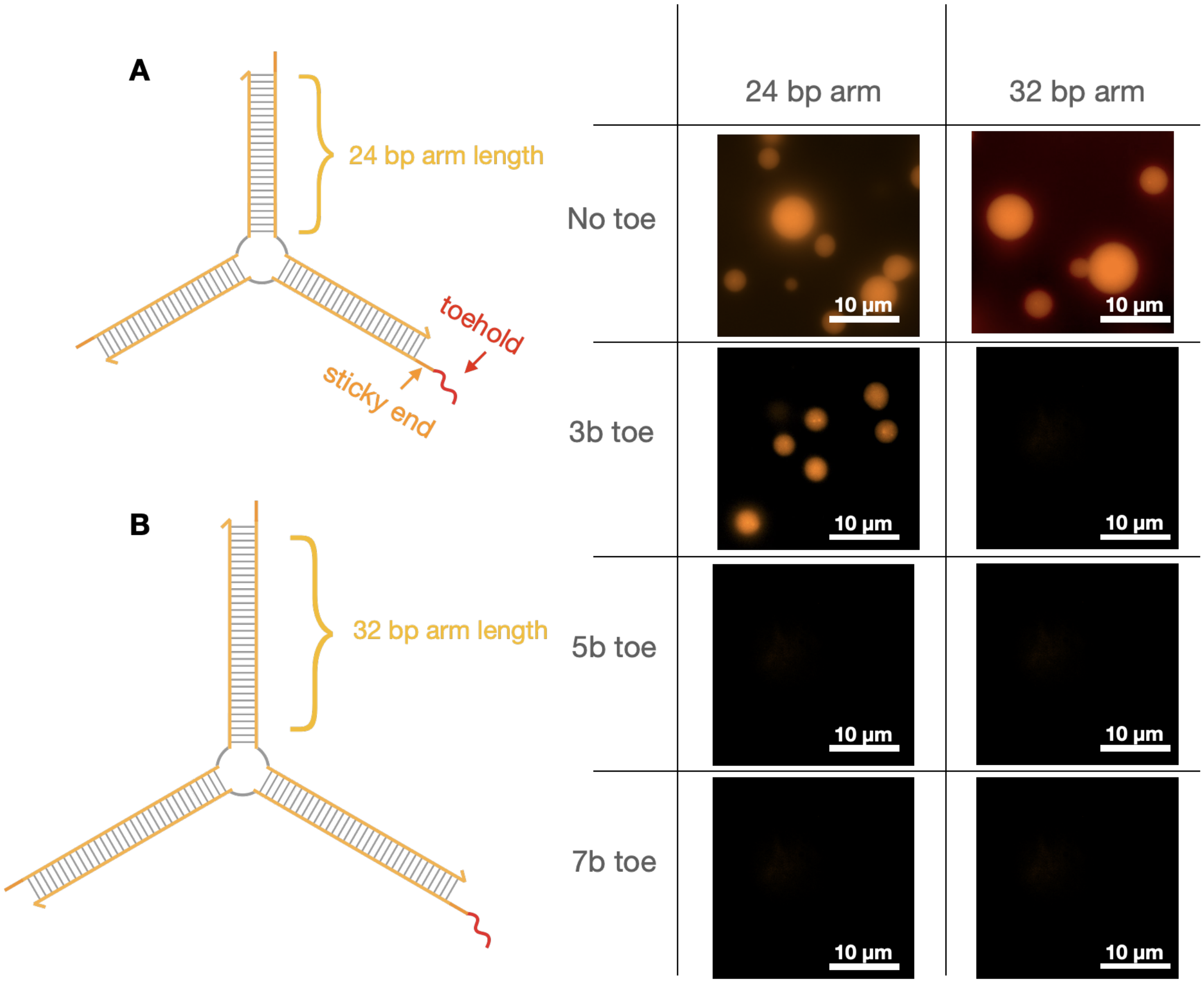

Supplementary Figure 2: Effect of toehold on NS featuring long arms. A: 24 bp arm and B: 32 bp arm designs with a toehold for invasion with different lengths of toehold. Condensates do not form when introducing 5 and 7 base long toeholds. Microscopy images representing the condensates after 30 mins of incubation at room temperature (27° C) after anneal process. Scale bars as represented.

2.3 Six arm nanostars with adjacent and staggered invasion points after 24 hours

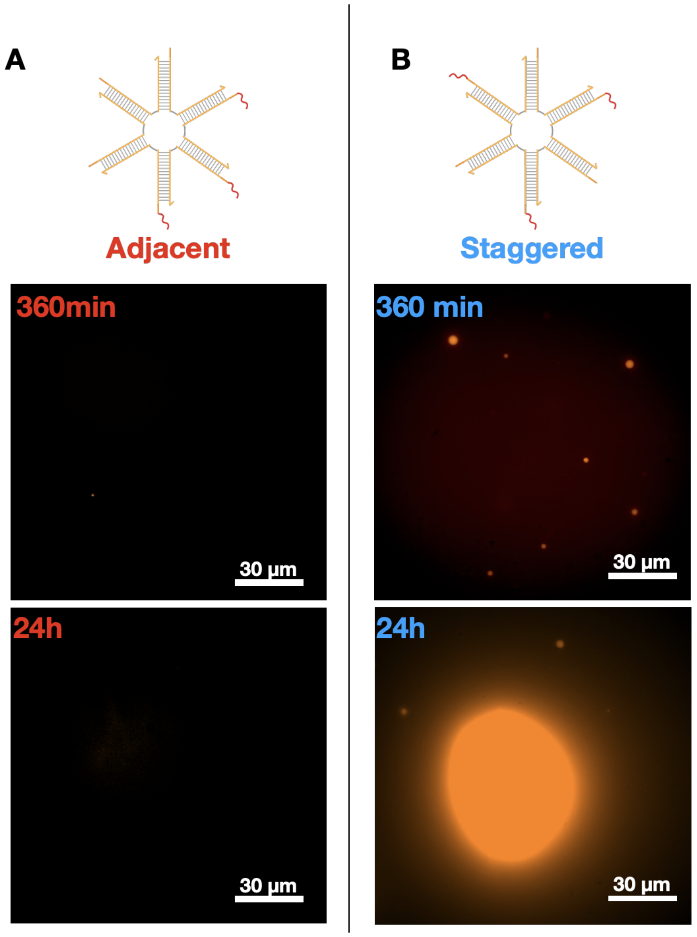

Supplementary Figure 3: DNA condensates formation using 6 arm nanostars including 3 toeholds for invasion. A: Adjacent toehold design and B: Staggered toehold design. Each panel includes representative microscopy images of the sample after 360 mins and 24 h incubation after invader addition at room temperature (27° C). [NS] = 5 µM, [I] = 5 µM (or 1X NS). Scale bars are 30 µm.

2.4 Orthogonal DNA condensates

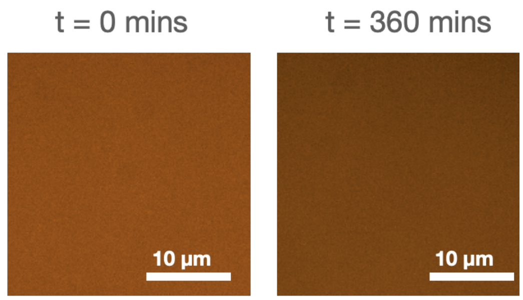

Supplementary Figure 4: Palindromic 4 nt sticky-ends containing only 'A's and 'T's do not yield condensates. Nanostars with 16 bp arms and a TATA Sticky end do not form condensates after 6 hours of incubation. Microscopic images represent time after 30 mins of incubation at room temperature (27° C) after anneal process. [NS] = 5 µM. Scale bars as represented.

2.5 Invasion and anti-invasion of orthogonal 6-nt NS at RT

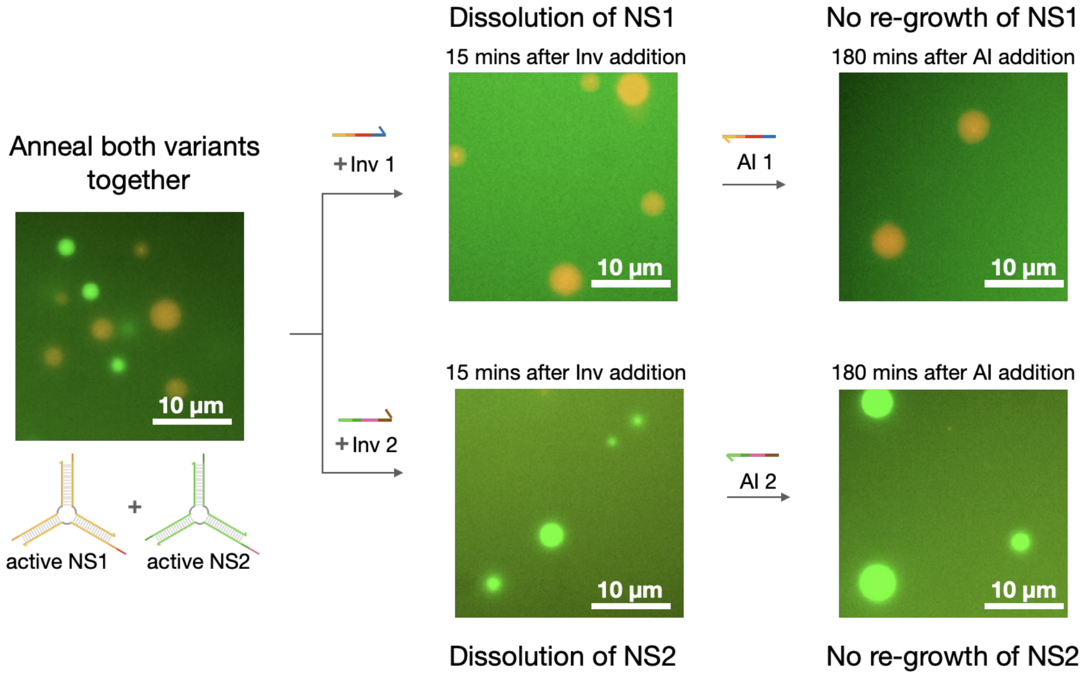

Supplementary Figure 5: Invasion and Anti-invasion of 6-nt NS at RT (27°C). Orthogonal Nanostars with 16 bp arms and a 6 nt Sticky end can be dissolved after invader addition within 15 minutes but do not regrow even after 3 hours of anti-invader addition. [NS] = 5 µM, [I] = 5 µM, [AI] = 5 µM. Scale bars as represented.

2.6 Excess of anti-invader limits droplet regrowth due to weak interactions with the nanostar sticky-ends.

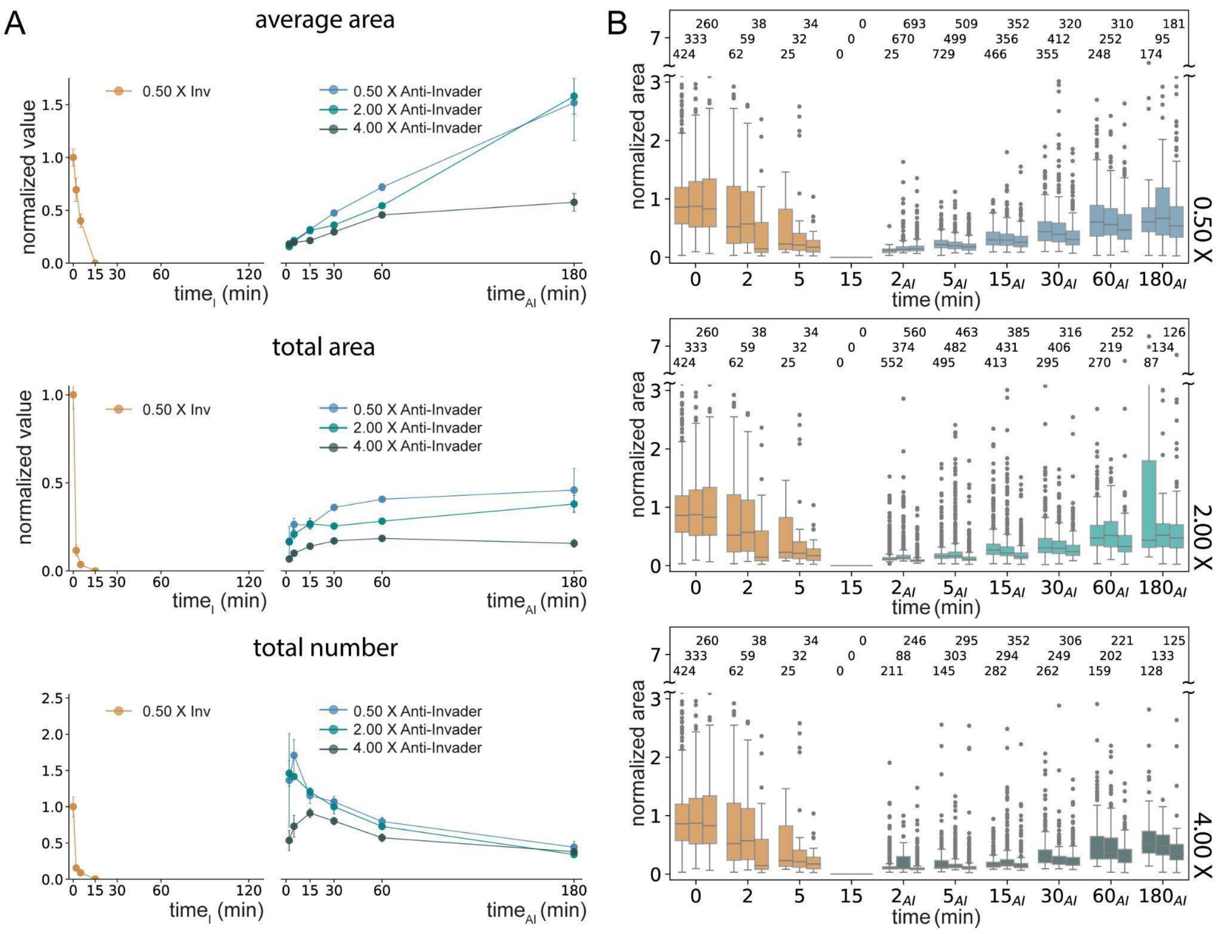

Supplementary Figure 6: (A) Normalized average area following addition of 0.5 X invader strand and adding different concentration of anti-invader strands. (B) Box plots illustrating droplet growth in the absence of invaders and with the addition of anti-invaders. In each box, the central line indicates the median, the bottom and top edges of the box indicate the 25th and 75th percentiles. Whiskers extend to the most extreme data points not considered outliers. Crosses indicate outliers. Excess of anti-invader limits droplet regrowth due to weak interactions with the nanostar sticky-ends.

2.7 Condensate regrowth in 4 arm NS with addition of more than one Anti-invader

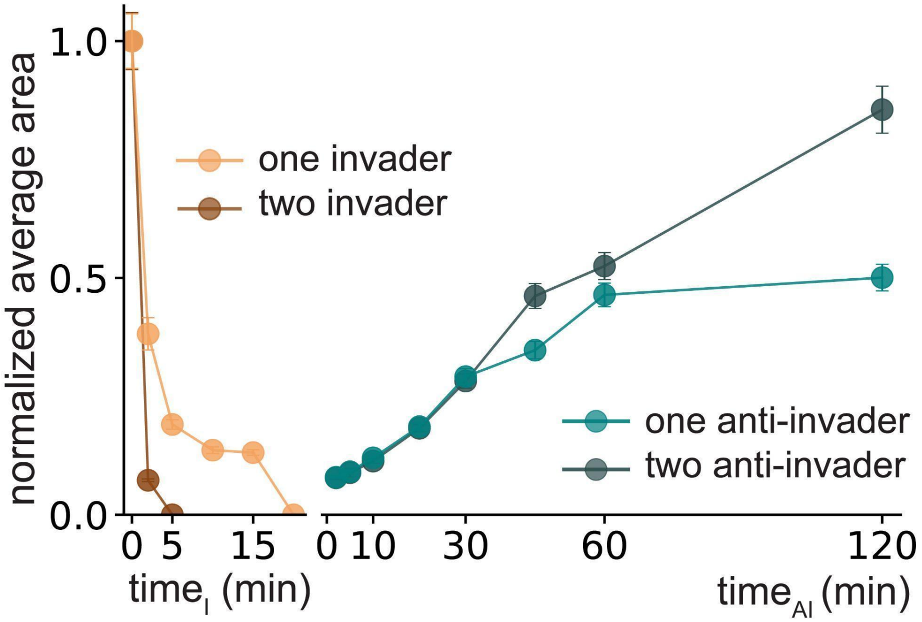

Supplementary Figure 7: Normalized average area following addition of either one or two invaders for the 4 arm NS design at 0.5X and adding respective anti-invader strands. The anti-invasion process for both cases do not have much of a difference up to 60 min. Data corresponds to a single experimental replicate. Error bars were obtained by bootstrapping.

2.8 Annealing nanostars in the presence of invader

The figures reported in this section complement the data reported in Figure 3 of the manuscript. As before, we consider [NS]=5μM annealed in the presence of different concentrations of invaders as reported in the captions. We report histograms of the droplet diameter, normalized average droplet area, total droplet area, and droplet number over time.

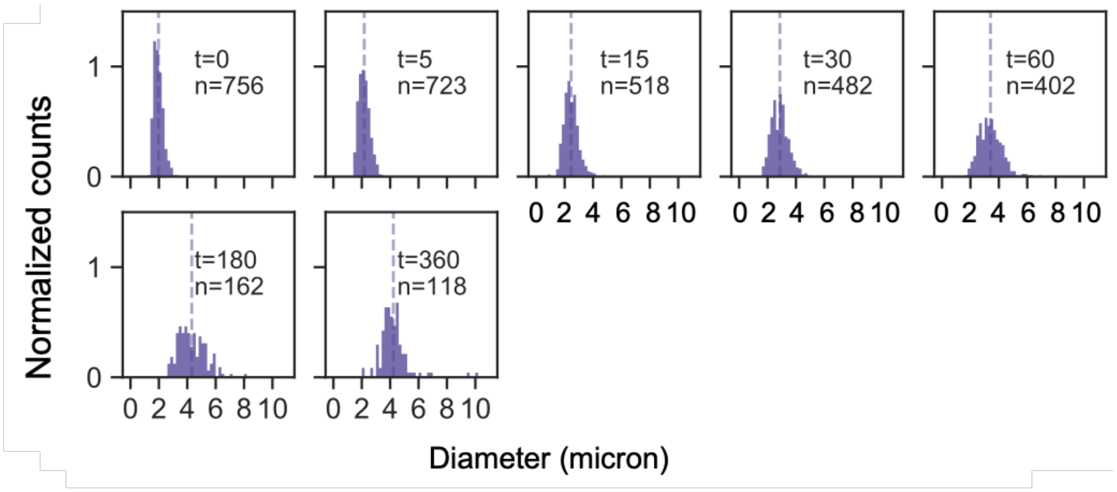

Supplementary Figure 8: Normalized histograms for simultaneous anneal of [NS] = 5 μM, [Inv] = 1.25 μM (or 0.25X NS). Histograms are normalized such that the area of each histogram is 1. Bin width is set to 0.2 μm. Vertical dashed line indicates the mean for each observed time. Data corresponds to a single experimental replicate.

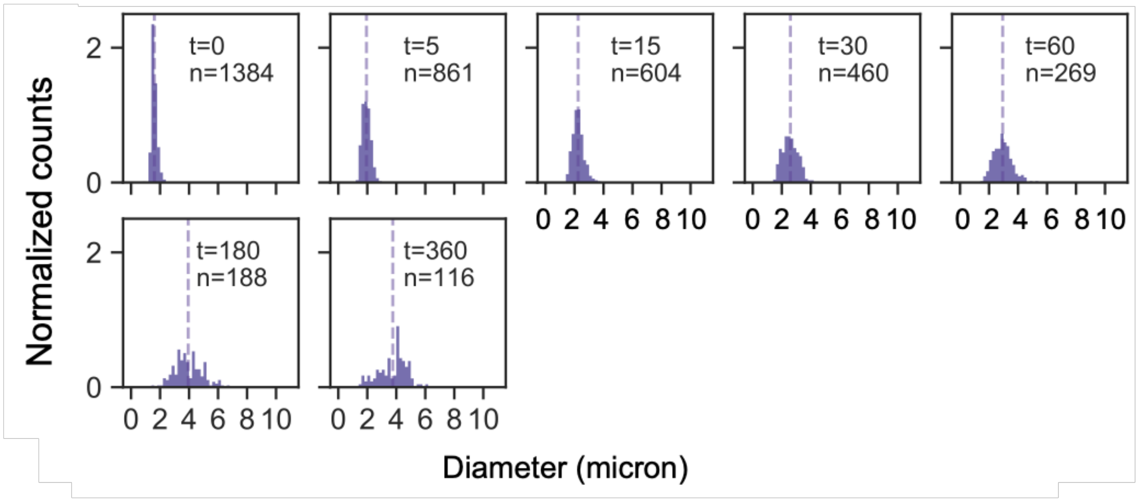

Supplementary Figure 9: Normalized histograms for simultaneous anneal of [NS] = 5 μM, [Inv] = 1.875 μM (or 0.375X NS). Histograms are normalized such that the area of each histogram is 1. Bin width is set to 0.2 μm. Vertical dashed line indicates the mean for each observed time. Data corresponds to a single experimental replicate.

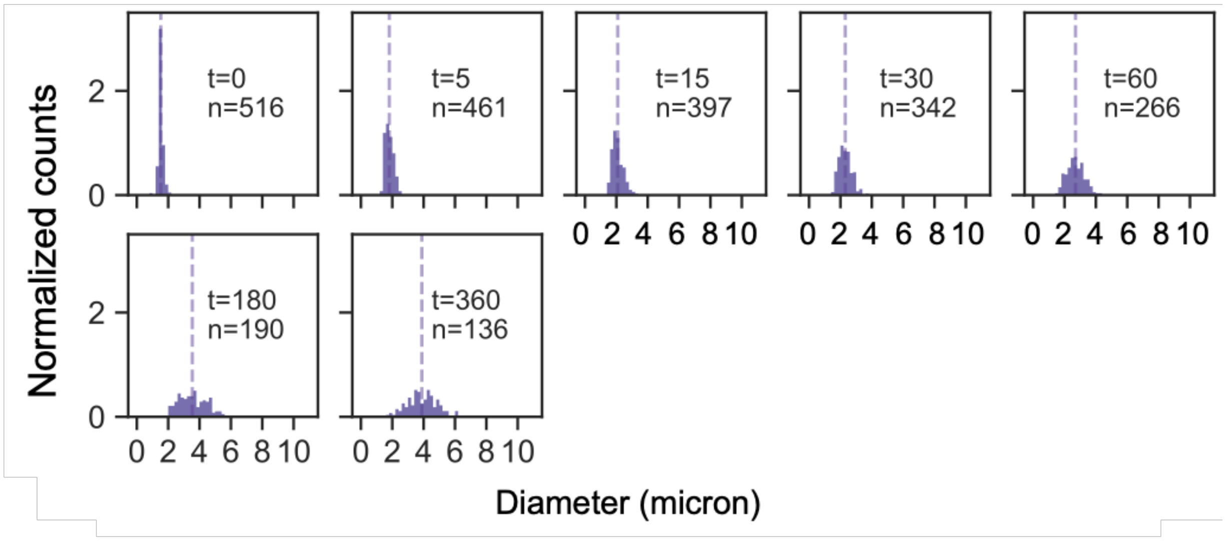

Supplementary Figure 10: Normalized histograms for simultaneous anneal of [NS] = 5 µM, [Inv] = 2.25 µM (or 0.45X NS). Histograms are normalized such that the area of each histogram is 1. Bin width is set to 0.2 µm. Vertical dashed line indicates the mean for each observed time. Data corresponds to a single experimental replicate.

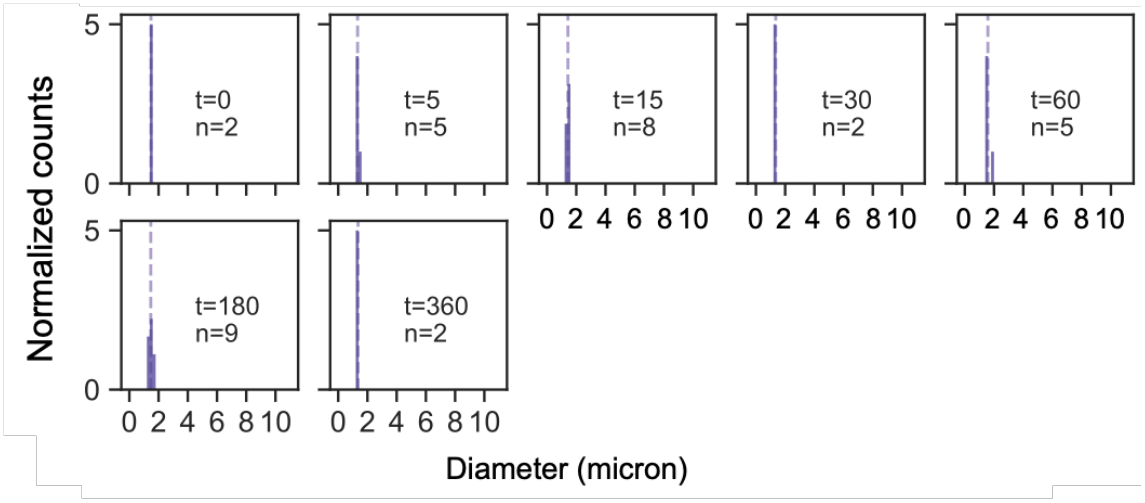

Supplementary Figure 11: Normalized histograms for simultaneous anneal of [NS] = 5 µM, [Inv] = 2.5 µM (or 0.5X NS). Histograms are normalized such that the area of each histogram is 1. Bin width is set to 0.2 µm. Vertical dashed line indicates the mean for each observed time. Data corresponds to a single experimental replicate.

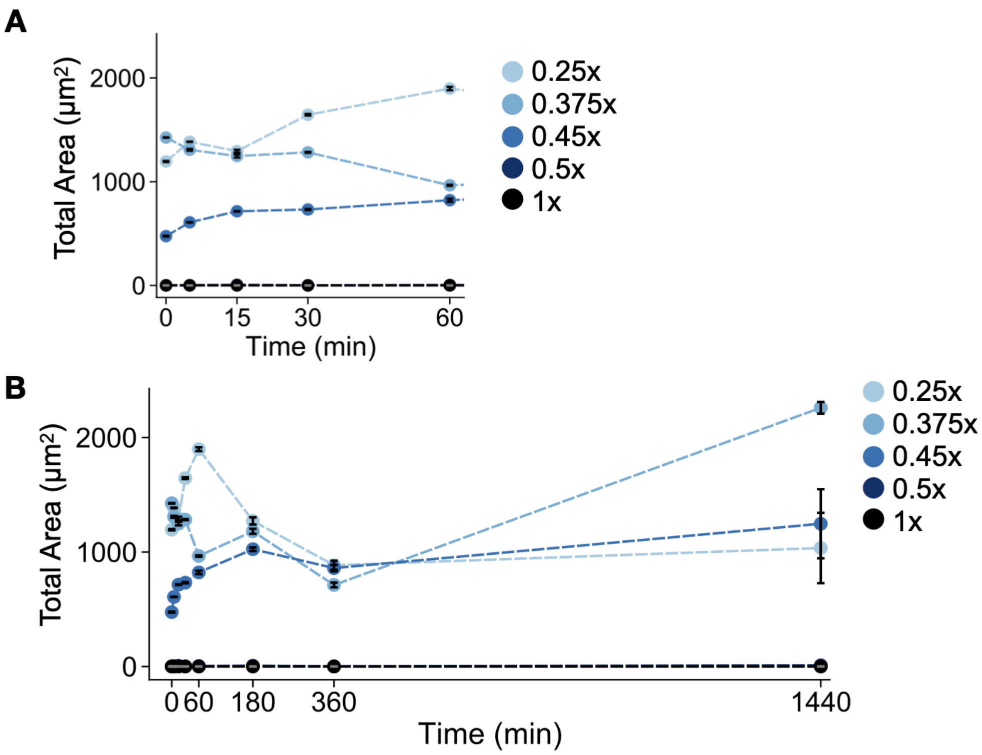

Supplementary Figure 12: Total area of condensate for nanostars annealed with different concentrations of invader strand through 1 hour (A) and through 24 hours (B). Data corresponds to a single experimental replicate. Error bars were derived from bootstrapping.

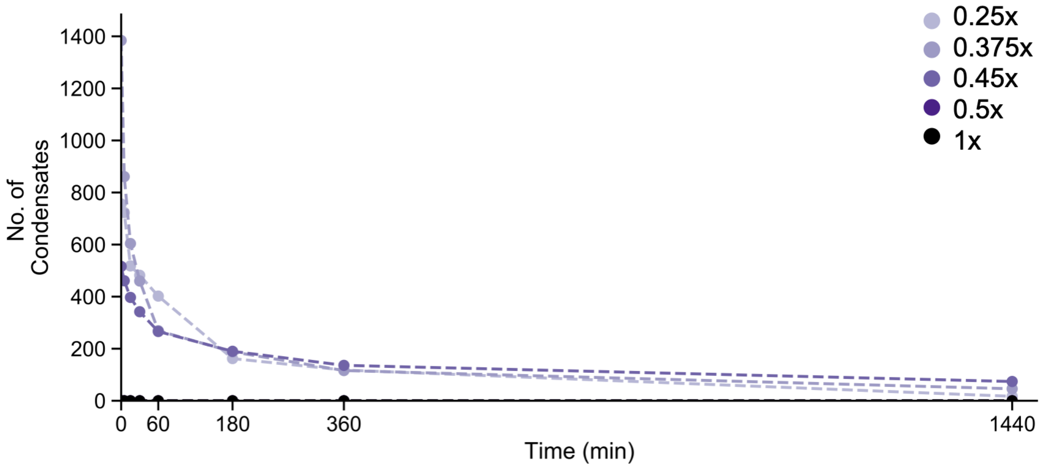

Supplementary Figure 13: Number of condensates, [NS]=5 µM, annealed with different concentrations of invader strand. Data corresponds to a single experimental replicate.

2.9 Annealing nanostars at different concentrations (no invader)

The figures reported in this section complement the data reported in Figure 3 of the manuscript. We consider DNA nanostars annealed at different concentrations. We report histograms of the droplet diameter, normalized average and total droplet area, and droplet number over time.

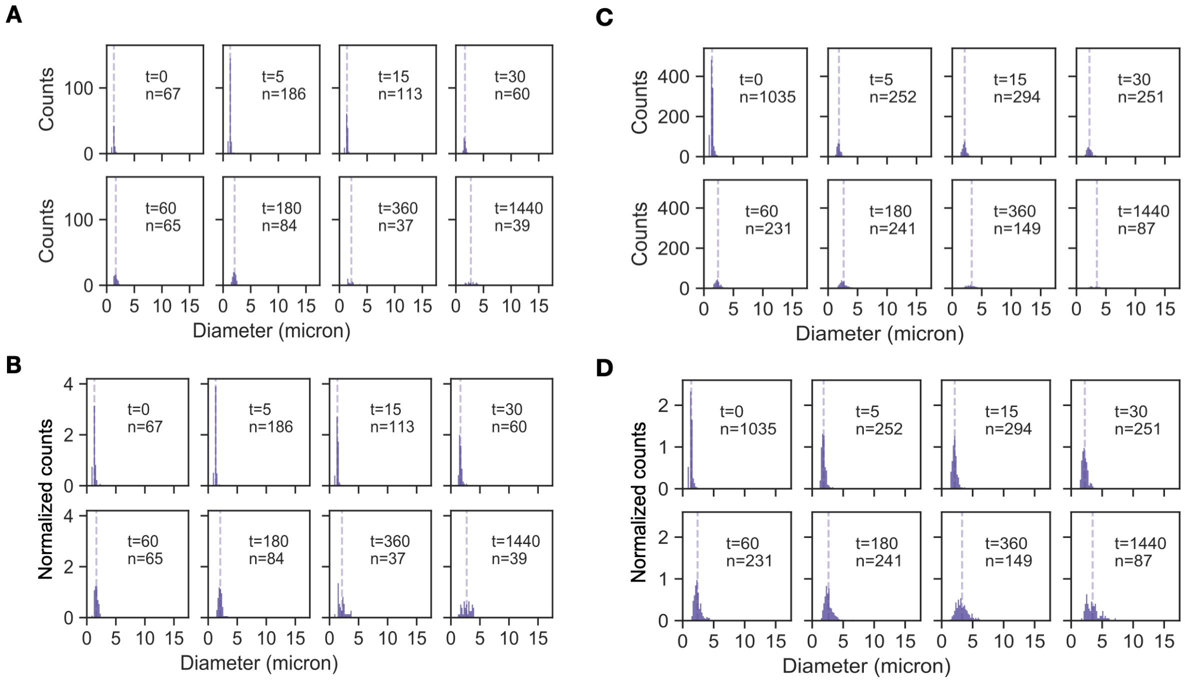

Supplementary Figure 14: Histograms and normalized histograms for 16-arm nanostars, [NS] = 0.25 μM (AB) and [NS] = 1 μM (CD). Histograms are normalized such that the area of each histogram is 1. Bin width is set to 0.2 μm. Vertical dashed line indicates the mean for each observed time. Data corresponds to a single experimental replicate.

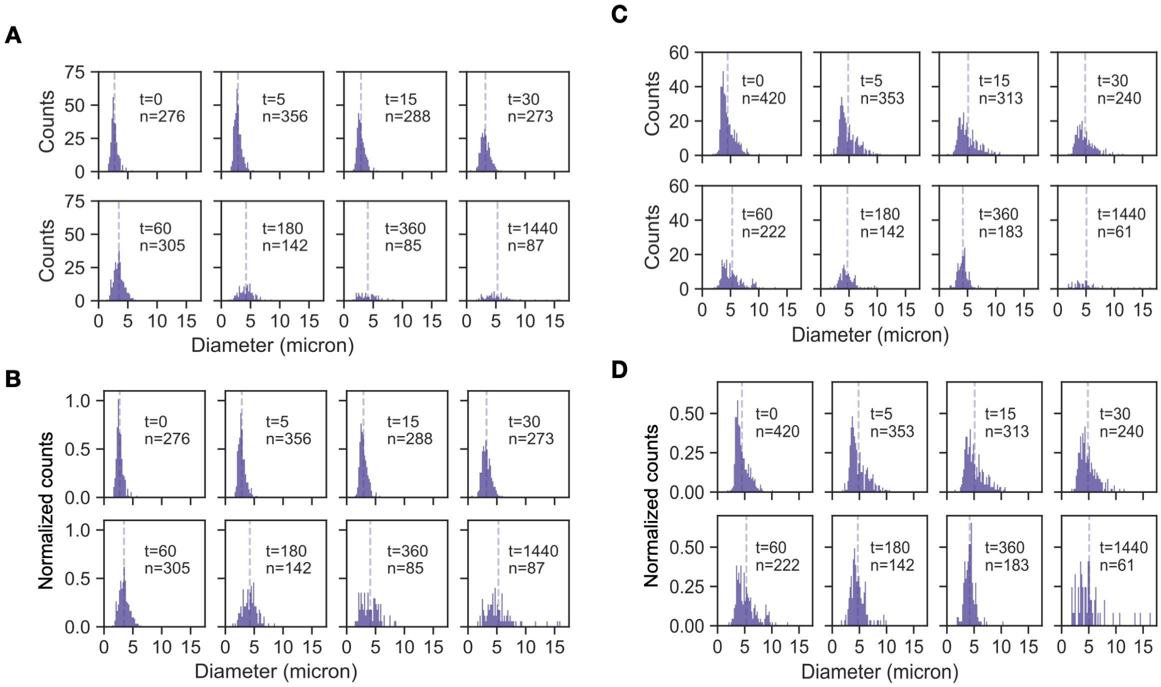

Supplementary Figure 15: Histograms and normalized histograms for 16-arm nanostars [NS]= 2.5 μM (AB) and [NS]= 5 μM (CD). Histograms are normalized such that the area of each histogram is 1. Bin width is set to 0.2 μm. Vertical dashed line indicates the mean for each observed time. Data corresponds to a single experimental replicate.

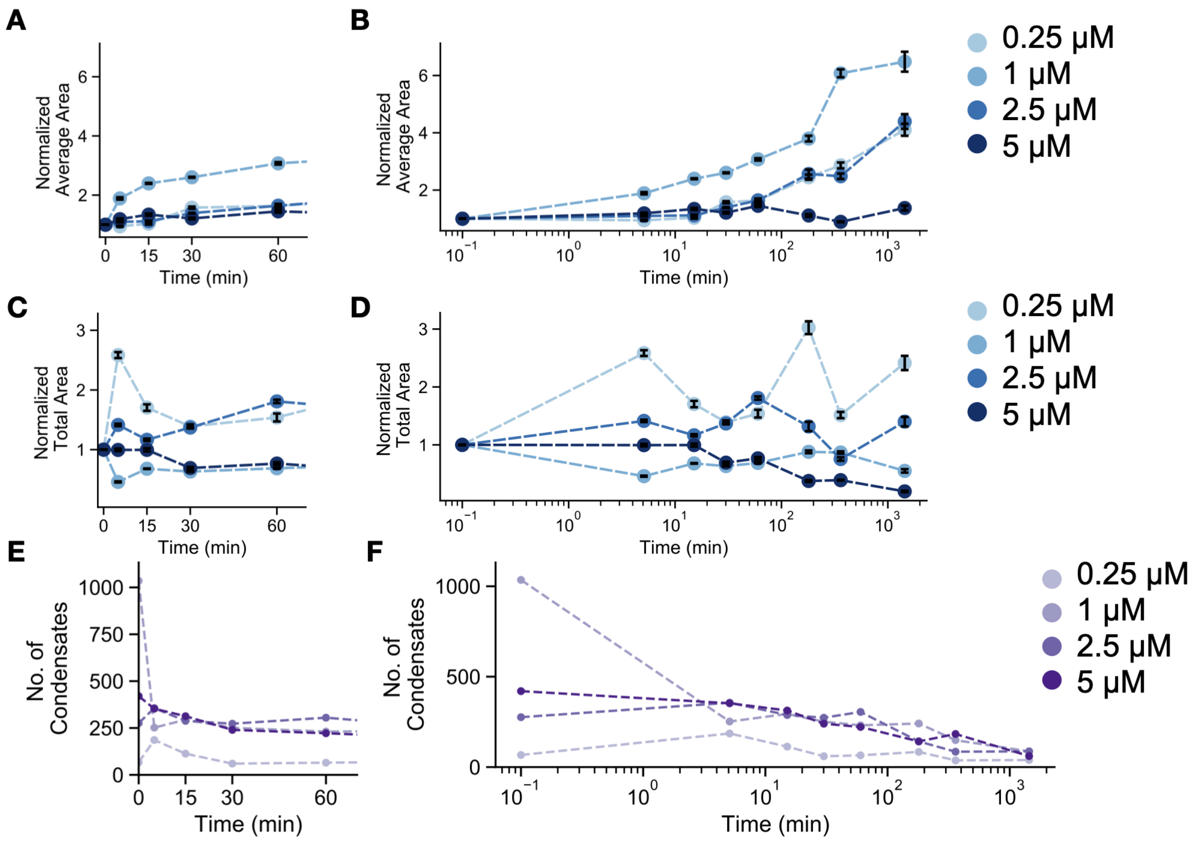

Supplementary Figure 16: Further characterization of 16 arm NS annealed and incubated without invader at different concentrations. Normalized average area of each condensate through 1 hour (A) and 24 hours (B). Normalized total area of condensate through 1 hour (C) and 24 hours (D). Both normalized average and total areas are normalized with respect to the initial value prior to addition of invader. Number of condensates for NS annealed with different concentrations of invader strand through 1 hour (E) and 24 hours (F). Data corresponds to a single experimental replicate. Error bars in A-D were derived from bootstrapping.

3. Supplementary Notes

Supplementary Note 1: Mean Field Theory Model

In this section we introduce the mathematical background behind the construction of the model used in the main text. We introduce a free energy to account for the phase separation aspect of the dynamics. For this purpose, we use the Cahn-Hilliard free energy, which is standard in phase separating systems

$$F = \nu([n]-c_0)^2([n]-c_1)^2 - \lambda^2(\nabla[n])^2 + \chi[n]^2[n_0]^2.$$

The first part is the standard Cahn-Hilliard free energy for phase separation. By itself it describes the fact that a system of phase separating subunits can undergo phase separation
We supplement this with an additionally coupling between the active and inactivated subunit with a coupling constant χ. Note that we couple the fields by $\chi[n]^2[n_0]^2$ and not $\chi[n][n_0]$ as the latter would include the effect of a field with initially no inactivated monomers spontaneously producing inactivated monomers recruited from the bulk The second order nature allows for local density conservation, i.e., if we start with a field with no inactivated subunit, after a short amount of time the subsequent field will also be zero everywhere (in the absence of chemical reactions). However, it is seen that none of the properties we are interested in depend critically on this parameter, it merely accounts for the fact that there is an effective repulsive interaction due to steric effects between the inactive and active subunit.
The dynamics of the system in the presence of chemical reactions can be obtained using the hybrid model AB dynamics[1].

$$\frac{\mathrm{d}\mathbf{x}}{\mathrm{d}t} = D\nabla^2\left(\frac{\delta F}{\delta \mathbf{x}}\right) + \mathbf{R}(\mathbf{x})$$

we make the assumption that the diffusion constant D for all the molecules in the material is the same, for simplicity. The term $\mathbf{R}$ arises from the mass action kinetics corresponding to chemical reactions of inhibition and activation presented in the main text. As this term does not arise from the free energy, the model described is inherently non-equilibrium, as it assumes that the inhibition and activation reactions proceed at some rate rather than defining the energies of the inhibited and activated molecules. We do not expect this to matter for the phenomena we seek to address, as we are not interested in the equilibrium behavior but the response of a non-equilibrated steady state to the introduction of additional chemical elements. (Additionally, in the experimental part of our work we are working in a regime where the reactions are essentially irreversible.)
The parameters of the free energy F, which were used to generate the plots in Figure 1 of the manuscript, are $\nu = 1$, c_0= 0.2, c_1= 0.9, λ= 0.6 and χ=0.
To investigate the dynamics, we used a nominal diffusion coefficient D=10 for the inhibitor and $D = 1$ for the monomers.

To generate the phase diagram in Figure 3 of the manuscript we used the same parameters as above, but with a non-zero, variable coupling constant χ.
In the main paper we consider the reaction term R(x) arising from the introduction of the inhibition and activation reactions. For the inhibition reaction:

$$n + I \underset{k_b}{\overset{k_f}{\rightleftharpoons}} n_0$$

We don't treat the finite valency found in DNA nanostars, instead the invading molecule completely inactivates the phase separating molecule from participating in phase separation. One could imagine suitable modification of the theory to include the effects of valency by using a more general Flory-Huggins theory with multiple phase separating elements, however in the present model we are most interested in expounding upon the most generic behavior.

Computational Integration

Our mean field equations are solved in Fourier space with periodic boundary conditions using a semi-implicit scheme with in-house C++ code apart from the Fast Fourier Transforms, which are performed using the FFTW library. Analysis of the areas was performed in *Wolfram Mathematica*.

Effective parameters underpinning Chemical Dissolution

The models we have described are non-equilibrium, therefore in order to analyze the static properties of these systems, we shall proceed by analyzing whether the homogeneous state is stable to small perturbations. The homogeneous state arises from considering the fixed points in the chemical dynamics. Around this state we apply a wavelength dependent perturbation:

$$\phi_i(\mathbf{x}) = \phi_{i0} + \delta \exp(\beta t - i\mathbf{k}.\mathbf{x})$$

for a small perturbation δ around the homogeneous value ϕ_{i0}. By studying how the growth rate β is different for different wave vectors $\mathbf{k}$ we can answer (to first order) whether the homogeneous state is stable. By looking at the system for different parameters, therefore, we can generate a "phase diagram" of the system in the presence of chemical reactions.

Furthermore, we can study the dynamical properties of the system by numerically solving the equations outright, however, prior to doing this it is worth reasoning over the possible dynamics we might expect to arise from chemical disruption
There are two different dynamical factors to consider for droplets in this case, growth and decay. It is easy to see that the nature of the growth process is not affected by the attacking chemical, i.e., we should expect the same kinds of coarsening dynamics of droplets to occur whether there is chemical inhibitor or not, as if we introduce chemical inhibitor which is not sufficient to homogenize the system, it will merely inactivate a certain proportion of the monomers, leaving the rest intact. One could then consider the subsystem containing only the active monomers, which would then be identical to the coarsening dynamics of the same system without inhibitor, under the caveat that there will be a small additional effect due to steric repulsion.
On the other hand, the process by which droplets decay away has the potential for novel phenomena. In contrast to the standard process of evaporation, which occurs from the surface, the case with an invasive species could have qualitatively different behavior depending on how

much the invading species can penetrate into the droplet before it inactivates it. This is something which is qualitatively different dynamically to the ordinary process of raising the temperature.
Doing dimensional analysis with this in mind, we wish to consider two different time scales. One timescale is the diffusion time for an inhibitor inside the droplet:

$$\tau_D = \frac{R^2}{D}$$

where R is the effective droplet size and D is the diffusion constant of the inhibitor inside the droplet. This is the only way we can obtain a timescale from a length and a diffusion constant which has dimensions $[\mathrm{Length}]^2[\mathrm{Time}]^{-1}$ This parameter roughly characterizes how long it would take for the inhibitor to diffuse a distance R in the droplet. To supplement this we need to consider how quickly the invading reactions occur. We characterize that with the following timescale:

$$\tau_R = \frac{1}{rc_1}$$

where r is the rate of reaction of conversion from a phase separating species and inactivating species into an inactivated complex, which would have the following equation in mass action kinetics:

$$\frac{d[I]}{dt} = r[n_M][I]$$

The dimensions of the rate are given by $[\mathrm{concentration}]^{-1}[\mathrm{time}]^{-1}$. The concentration used in the time scale is the concentration of the phase separating material in the droplet c_1
We postulate that the effective qualitative differences in evaporation scenarios arise from the dimensionless parameter:

$$\delta = \frac{\tau_D}{\tau_R}$$

From which we can identify different regimes as a function of this parameter. For instance if $\delta << 1$ then the diffusion time is much shorter than the reaction time, and we would expect that the inactivator principally acts at the surface of the droplet. By contrast, if $\delta >> 1$ The inhibitor can diffuse freely through the droplet before it has even had time to react and we might therefore expect that the subsequent dynamics depend more on the volume of the droplet. In full this parameter is given by:

$$\delta = \frac{rc_1 R^2}{D}$$

In a simulation R^2 is a result, not something we can tune. However, all the other parameters we control.
If equilibria depends on $r\rho_{I_0}$ then we can tune dynamical response by keeping this factor fixed while we change D. In actuality, our system has a natural length scale λ, given by the surface tension, leading to:

$$\delta_\lambda = \frac{rc_1\lambda^2}{D}$$

Non-dimensionalizing the model equations directly leads to the appearance of parameter δ_λ (not shown). Depending on the regime in which the system is in, the timescales τ_D and τ_R tell us how quickly they should proceed. So while rescaling both by a factor a should leave both unchanged, the process itself should be sped up by a factor a.

Supplementary Note 2: Impact of Reverse Reactions

The reactions considered in the main text are:

$$n + I \underset{k_b}{\overset{k_f}{\rightleftharpoons}} n_0$$

$$n_0 + A \underset{k_b}{\overset{k_f}{\rightleftharpoons}} n + W$$

For kinetic simulations, we set the backwards rate to zero. The primary reason the analysis was performed in this regime was that we wanted to observe the “bare” effect associated with chemical dissolution, and not convolute it with additional factors corresponding to reverse rates (which should promote growth of droplets when the inhibition reaction is reversed, and vice versa for the activation reaction.

In this section we present supplementary Supplementary Figure 62, which illustrates the effect on the phase diagram of considering reverse rates in our system.

We can observe that a change in the relative reaction rates leads to an interpolation between two limiting behaviors, which is a stripe of phase separation justified to the right, and a stripe of phase separation going straight upwards (as a function of [I]). Both of these cases can be readily understood physically. The former arises from the fact that in the absence of inhibitor, the system undergoes phase separation within some region of concentrations $c_i < c^* < c_o$, and, to first order, when we add inhibitor we merely deactivate a proportion of the monomers (forever in the case of an irreversible reaction), such that the new condition would appear as $c_i < c^* - [I] < c_o$ depending on the stoichiometry of the inhibition. This would lead to the stripe of the phase diagram heading to the right. The latter case, where the reverse rate is very large, is also simply understood from the idea that if the reverse rate is infinite, there is no inhibitor effect. The preceding discussion ignores realities such as steric repulsion and additional interactions between the participating elements, however, we do not expect that the qualitative behavior should change greatly as a function of this.

The regimes in between these two consist in what appears to be an interpolation between these two limits. Interestingly, in the case that the rates are comparable to one another, we obtain a phase diagram that strongly resembles liquid-liquid phase separation with concentration on the x-axis and temperature T on the y-axis. This is suggestive that similar control over droplets that can be obtained via temperature could be obtained through the action of inhibitive chemical dynamics, but without changing temperature itself (which is a very all encompassing control parameter).

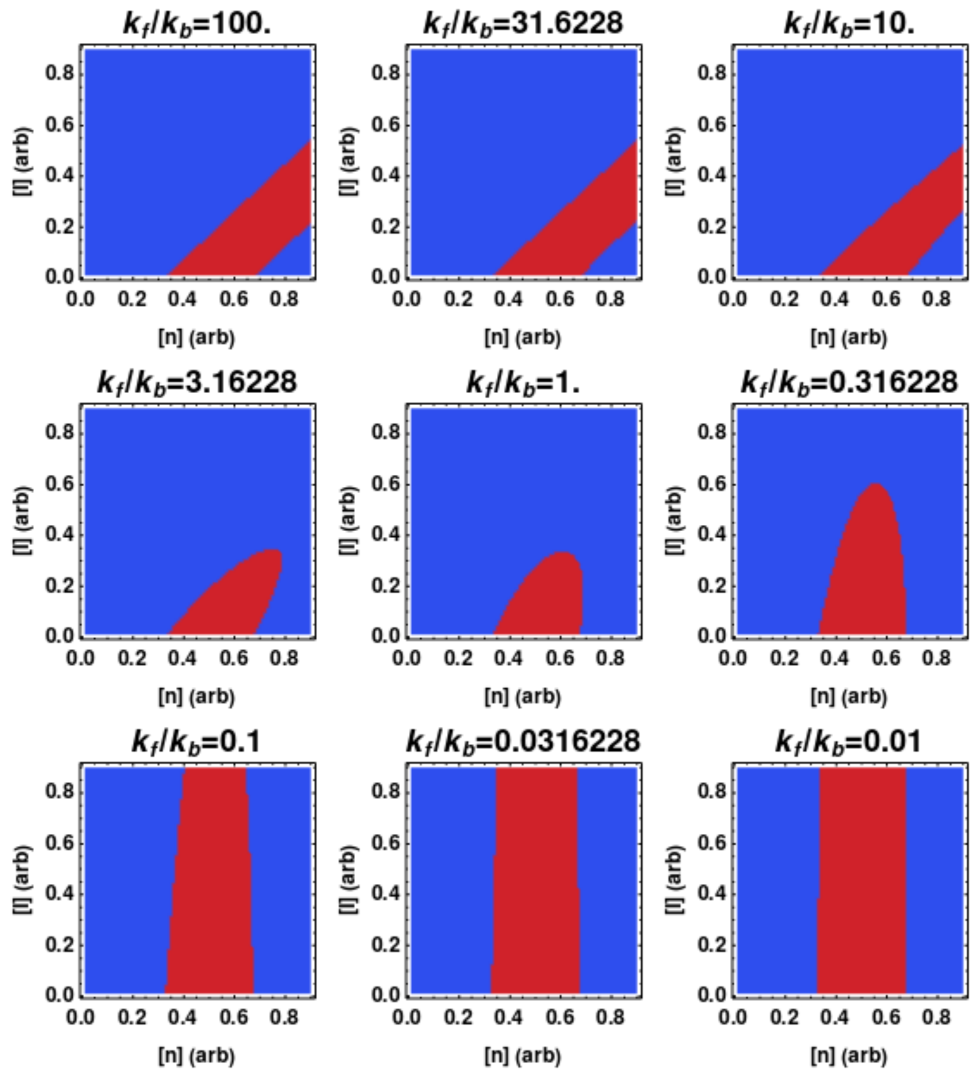

Supplementary Figure 17: Phase diagrams for inhibition when we allow a reverse reaction of an inactivated monomer spontaneously turning back into an active monomer plus inhibitor. In all plots, we show the region where the system undergoes phase separation in red, and where the homogeneous state is the most stable in blue. The diagrams are plotted against concentration of monomer [n] and the concentration of inhibitor [I] with the choice of dilute and dense concentrations for the monomer as 0.2 and 0.8, respectively. It can be seen that modification of the relative magnitudes of the rates leads to rather different phase diagrams in [n]-[I] space.

Supplementary Note 3: Other features

These simulations complement the results shown in Figure 1 of the manuscript. Figure S63 illustrates how depending on the system parameters it may be possible for droplets to regrow at low amounts of inhibitor, which introduces only a kinetic (transient) dissolution. In Figure S64 we show example images corresponding to volume driven or surface driven dissolution. In Figure S64 we include simulations showing the lack of dependence of regrowth on the diffusion constant of activator, and the timescale of inhibition relative to droplet size.

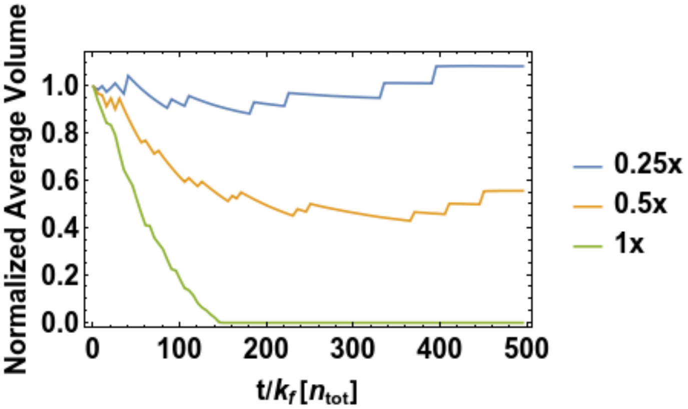

Supplementary Figure 18: Addition of inhibitor at different concentrations over long timescales. 0.25x and 0.5x are not sufficient to move the system to a region where droplets are no longer thermodynamically favored, therefore the droplets will begin to regrow over long timescales. $c_0 = 0.1, c_1 = 1.0, \lambda = 0.2, [n] = 0.4$, where $[n]$ is the concentration of phase separating monomer

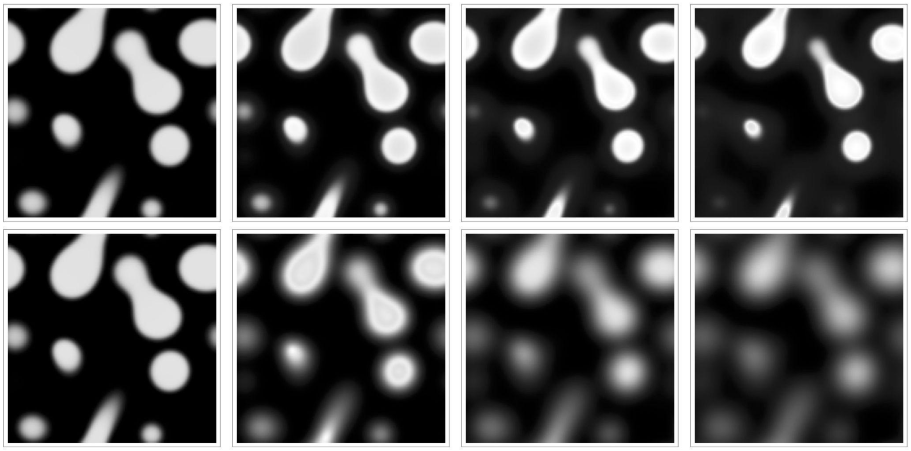

Supplementary Figure 19: Different mechanisms of dissolution for droplets, as one changes the diffusion constant of the inhibitor, the droplets will either slowly decay from their surface (top), or the deactivation process occurs throughout the volume of the droplet (bottom). In the former case, we observe the droplets becoming smaller in size, but retaining their shape. In the latter, we observe a “smearing” of the droplets. Parameters: $c_0 = 0.2, c_1 = 0.9, \lambda = 0.6, [n] = 0.4$, where $[n]$ is the concentration of the phase separating monomer.

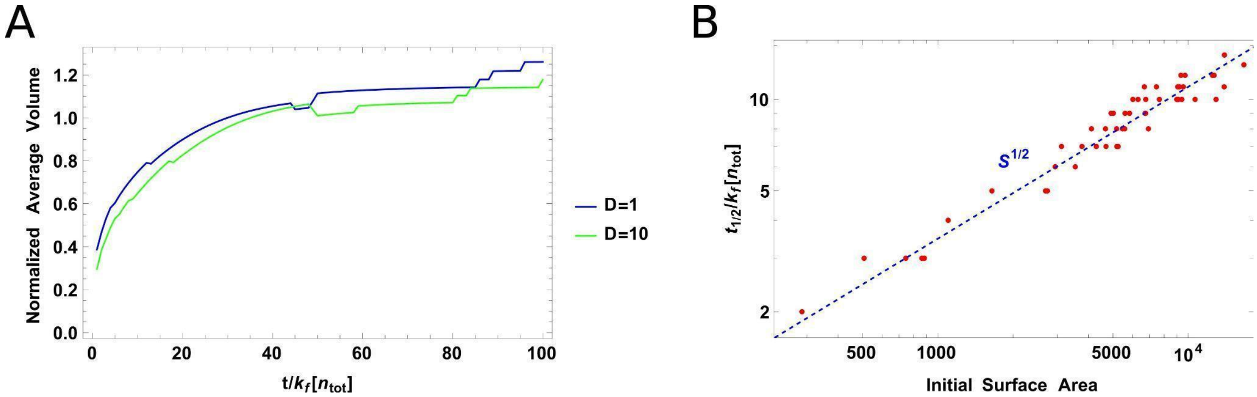

Supplementary Figure 20: A) Regrowth of droplets under addition of activator having different diffusion constants; this parameter does not affect the speed of regrowth. $c_0 = 0.2, c_1 = 0.9, \lambda = 0.6, [n] = 0.4$ B) The half-life: time until a droplet reaches half its size scales to the 1/2 power of the surface area S of the droplet. $c_0 = 0.1, c_1 = 1, \lambda = 0.2, [n] = 0.4$

4. Supplementary References

1. Hohenberg, P. C. & Halperin, B. I. Theory of dynamic critical phenomena. *Rev. Mod. Phys.* **49**, 435–479 (1977).
